# Mathematical analysis for spatial distribution of vessels, mast cells and adipocytes in superficial fascia

**DOI:** 10.3389/fphys.2022.1026019

**Published:** 2022-11-14

**Authors:** Yingyue Dong, Dandan Zhang, Yingri Cao, Yanfei Zhang, Xiaozhe Sun, Tongsheng Chen, Yuanyuan Zhang, Guoheng Xu

**Affiliations:** ^1^ Department of Physiology and Pathophysiology, School of Basic Medical Sciences, Peking University, Beijing, China; ^2^ Department of Civil Engineering, Tsinghua University, Beijing, China; ^3^ Department of Anatomy, School of Chinese Medicine, Beijing University of Chinese Medicine, Beijing, China; ^4^ Key Laboratory of Functional and Clinical Translational Medicine, Department of Physiology, Xiamen Medical College, Xiamen, China

**Keywords:** fascia, blood vessel, mast cell, adipocyte, spatial pattern, correlation

## Abstract

As a novel origin of adipocytes, the superficial fascia, a typical soft connective tissue, has abundant adipocytes and preadipocytes, accompanied by numerous mast cells. Blood vessels pass through the fascia to form a network structure. The more reasonable statistical analysis methods can provide a new method for in-depth study of soft connective tissue by clarifying the spatial distribution relation between cells (point structure) and blood vessels (linear structure). This study adopted the Guidolin et al. statistical analysis methods used by epidemiology and ecology to quantitatively analyze the distribution pattern and correlations among blood vessels, adipocytes, and mast cells. Image-processing software and self-written computer programs were used to analyze images of whole-mounted fascia, and the relevant data were measured automatically. Voronoi’s analysis revealed that the vascular network was non-uniformly distributed. In fascia with average area of 3.75 cm^2^, quantitative histological analysis revealed 81.16% of mast cells and 74.74% of adipocytes distributed within 60 μm of blood vessels. A Spearman’s correlation coefficient (rs) of >0.7 showed the co-distribution of the two types of cells under different areas. Ridge regression analysis further revealed the spatial correlation among blood vessels, adipocytes and mast cells. The combination of classical epidemiological analysis and extended computer program analysis can better analyze the spatial distribution relation between cells and vessels and should provide an effective analysis method for study of the histology and morphology of fascia and related connective tissues.

## Introduction

The superficial fascia, a typical soft connective tissue, is connected to the skin and the deep fascia, which extends continuously throughout the whole body (Stecco et al., 2011; Pirri et al., 2022a). Because the superficial fascia is located below the skin, it is usually tightly associated with or embedded in subcutaneous adipose tissue in humans. It plays an important role in holding, organizing, and storing adipose tissue (Nakajima et al., 2004; Abu-Hijleh et al., 2006; Pirri et al., 2022b). In fact, histological specimens show that the superficial fascia consists of collagen fibers and adipocytes and is pervaded by blood vessels (Pirri et al., 2022c; Fede et al., 2022). Our laboratory previously identified lineage-committed adipose precursor cells residing in the superficial fascia in rats. This finding supports that fascial adipocytes are a unique population of adipocytes and the superficial fascia is an origin of adipose cells (Su et al., 2016; Zhang et al., 2020).

In previous studies, we noticed that adipocytes were always accompanied by a large number of mast cells in superficial fascia (Zhang et al., 2019). Mast cells are widely distributed in fat, but the dense vascular network in adipose tissue is also an essential component. However, an important component, blood vessels, has been neglected in previous studies. In histology, mast cells are generally believed to distributed close to vessels (Abraham and St John, 2010). Mast cell precursor cells circulate in the blood, migrate from blood vessels and colonize in tissues, and become differentiated to mast cells (Yong, 1997). Adipocytes need nutrients provided by blood vessels and vascular stromal cells are also a source of preadipocytes (Cawthorn et al., 2012). However, quantitative analysis of blood vessels in adipose tissue in related research fields has always been lacking.

The relation between mast cells and adipocytes was confirmed in spatial distribution and cytological experiments in two other studies in our laboratory (Zhang et al., 2019; Chen et al., 2021), but they did not involve the dense vascular network on the superficial fascia. This study aimed to confirm the distribution pattern of blood vessels on the superficial fascia and the relation between the distribution of blood vessels and the two kinds of cells, demonstrating the spatial distribution relation between mast cells and adipocytes in the presence of blood vessels as an influencing factor and improving on previous conclusions. Because the size of two kinds of cells in superficial fascia differs greatly and the distribution of blood vessels is irregular, many statistical methods are difficult to use. Therefore, the computer-based morphometric analyses used in this study provided a novel insight into the histological quantification of the spatial connections between spot-shaped objects and linear-shaped structures, such as cells and vessels or nerves.

In macro epidemiology, methods are often used to analyze whether the occurrence of a disease is clustered and whether the disease is related to other diseases or environmental factors (Galrell, 1996). This is similar to the relation between cells and blood vessels. Guidolin et al. investigated the spatial distribution of mast cells around vessels and glands in human gastric carcinoma (Guidolin et al., 2004; Guidolin et al., 2017a; Guidolin et al., 2017b). We aimed to introduce the authors’ mathematical analysis methods into the study of biological histology of rat superficial fascia. By analyzing the panoramic patch, a complete analysis process judges the spatial distribution relation between cells, blood vessels and other structures. This study used a variety of methods to analyze the spatial distribution correlation: Voronoi’s analysis was used to analyze the distribution of the vascular network (Fortune, 1986; Marcelpoil and Usson, 1992; Guidolin et al., 2004). The distribution pattern of cells with respect to blood vessels was analyzed by comparing it with random distribution, referring to K function (Philimonenko et al., 2000; Guidolin et al., 2017a; Guidolin et al., 2017b), and the correlation between cell distribution was analyzed by Spearman’s coefficient (Zhang et al., 2019; Abd Al-Hameeda, 2022).

The study first covered the spatial distribution relation among mast cells, adipocytes and blood vessels. We first showed that blood vessels are randomly and disorderly distributed on the fascia. Then we measured the distance from all mast cells and adipocytes to blood vessels. On fascia with an average area of about 3.75 cm^2^, 81.16% of mast cells and 74.74% of adipocytes were distributed within 60 μm from blood vessels. The Spearman’s correlation coefficient (rs) between mast cells and adipocytes showed that the two cell types were co-distributed, and ridge regression analysis further confirmed the accuracy of this conclusion. We found a close spatial distribution correlation among mast cells, adipocytes and blood vessels, which may depend on their physiological functions. Also, the findings provide a unique technique for analyzing spatial and functional interactions *in vivo* and vice versa. They could provide useful information for research on the physiology and pathology.

## Materials and methods

### Animals

Male Sprague–Dawley rats were used in the study. Selected weaning rats were fed a standard chow diet for 4 weeks. Rats weighing 180–200 g are more suitable for preparing whole-mounted fascia slides. At this stage, the area of fascia is large enough for histological analysis, and the number of fascial layers is still low. Fascia slides for each individual experiment were obtained from three rats. Animal experiments were approved by the animal care and utilization committee of Peking University Health Science Center and conducted in accordance with the NIH guidelines for the care and use of laboratory animals.

### Preparation of whole-mounted fascia

Fascia is a type of loose connective tissue that forms a continuous structure covering the whole body. The fascia envelopes and separates organs, tissues and cells. Superficial fascia is located between the subcutaneous dermis and the deep fascia of skeletal muscles. The superficial fascia of the hind limb is easy to separate completely. In addition, because fat is more concentrated here, it will not interfere with the slide. The sheet of superficial fascia was lifted with surgical tweezers and cut with scissors, carefully avoiding damaging the integrity of the fascia during the separation process. Fascia samples were whole-mounted onto glass slides, and attention was paid to avoid excessive traction of the fascia. Whole-mounted fascia slides were air-dried at room temperature and then fixed in 4% paraformaldehyde for 15 min. Fascia slides were stored in PBS for temporary storage before staining (Zhang et al., 2019).

### Staining of mast cells

Toluidine blue was used for staining mast cells. The slides of whole-mounted fascial tissues were stained with 1 mg/ml toluidine blue for 1 min. Thionine was also used to show mast cells: slides were stained with 0.5% thionine for 5 min.

### Histological digital images of fascia slides

Superficial fascia slides should be panoramically scanned to obtain the most complete histological information. A panoramic digital slide scanner (3DHISTECH, Budapest) was used to scan slides of whole-mounted fascia. A CaseViewer digital microscope application supplied by the manufacturers was used for picture display and processing.

### Measurement of the distance between cells and the nearest blood vessel

For each mast cell in the fascia slide, the adjacent vessels were first found and the distance from the center of the mast cell to the adjacent vessels was measured, which is equivalent to measuring the distance from a point to a line. Then, the shortest distance was selected to define the distance between this mast cell and the nearest vessel. The distance from each mast cell to the nearest vessel was measured and recorded.

First, the center point of each mast cell and the traces of blood vessels in the image were marked manually on the image. Then the nearest distance from the center point mark of each mast cell to the traces of the blood vessel centerline was measured. In-house computer programs were used to perform repetitive and single operations in the distance measuring. According to the actual size corresponding to the 1-pixel length of the image, the distance from the center of each cell to the blood vessel was calculated.

This method used to measure the distance between adipocytes and the nearest vessel was consistent with the method used for mast cell measurements.

### Counting the number of cells within a certain distance from the vessel

The distance from each mast cell in the fascia slide to the nearest vessel was measured, and the data were sorted by distance from small to large. The mast cells with distance less than or equal to the set distance were directly screened, and their number was determined. Thus, we obtained the distribution of the nearest distance between all mast cells and blood vessels in a certain area.

### Analysis of vascular network structure

The program was performed according to the methods described by Guidolin et al. (2004). This method has been used in the study of angiogenesis to detect differences in the angiogenic response elicited by various factors and is useful for the objective quantitation of the vascular network structure.

This method simplifies the analysis of the morphology of the linear structure of blood vessels to the distribution of points. The branch points of blood vessels are extracted, and the vessel graph is simplified to a set of points. If the spatial pattern of branch points of vessels is regular, the vascular structure is ordered. Therefore, two types of order exhibited by branch points were considered: positional order and topological order (Kirchner et al., 1996; Smith et al., 1996).

(a) Positional order is associated with the arrangement of the network in the membrane. A Voronoi diagram, a classical method of geographic epidemiology, was used to analyze the spatial pattern of branch points of the blood vessels in the superficial fascia. In the classic case, British physician John Snow used a Voronoi diagram in 1854 to illustrate that cholera was spread by contaminated water pumps in Soho. The Voronoi image was drawn according to the branch points of blood vessels. The Voronoi image of the vascular network consists of the perpendicular bisector of the line segment between two adjacent branch points. If the set of points is distributed regularly, the area of each unit area in the generated Voronoi image should be the same. Therefore, the following parameter (ranging from 0 to 1) was estimated:

$$SD=1-{\left(1+\frac{\sigma_{A}}{\bar{A}}\right)}^{-1}$$

where $\bar{A}$ is the mean area of the Voronoi cells and $\sigma_{A}$ is the standard deviation.

In particular, with increasing heterogeneity, the SD (ranging from 0 to 1) also increases. The closer the SD value to 1, the more disordered the distribution of blood vessels.

(b) Topological order is associated with the homogeneity of branching occurrence along the structure. The lengths between the points where branching occurs (taken along the structure) should be equal if the vascular network structure is ordered. Therefore, we measured the length of the skeleton segments, and the following parameter was estimated:

$$TD=1-{\left(\;1+\frac{\sigma_{L}}{\bar{L}}\right)}^{-1}$$

where $\bar{L}$ is the mean length of the skeleton segments and $\sigma_{L}$ is the standard deviation.

This parameter increases with increasing heterogeneity in branching point occurrence. The larger the value of TD (ranging between 0 and 1), the more disordered the vascular network (Guidolin et al., 2004).

A new layer was added to Photoshop for images of whole-mounted fascia, and a paintbrush with a thickness of one pixel was used to trace the blood vessels. After importing images of blood vessels into the program, the program finds and marks the location of branch points of the blood vessels and generates a Voronoi diagram; then, L and A can be calculated. The program also displays the processed image. One must check whether the marking of the cross-point of the vessel is correct.

### Analysis of the relation between mast cell and vascular distribution

In general, spatial patterns of sets of points and lines can be classified as clustered around the line, clustered away from the line, or random (points exhibit an approximately Poisson distribution in space with no obvious spatial association about the lines) (Pfeiffer et al., 2008). To determine the spatial pattern of mast cells among blood vessels in the superficial fascia, we used the geographic epidemiology method (Galrell, 1996). This method is based on common methods of epidemiological study of spatial clustering of disease and was improved by Guidolin et al. to adapt the method to quantitative histological analysis (Guidolin et al., 2017a; Guidolin et al., 2017b).

First, the null hypothesis is simply that “no clustering exists,” which is random spatial dispersion. If the null hypothesis is true, there should be no difference in the distribution pattern of cells and random points. In this study, the cumulative distribution frequency of the distance from a cell and a random point to the blood vessel should be equal. If the two values are not equal, then the observed pattern differs significantly from the null hypothesis of complete spatial randomness. This indicates that the spatial distribution of cells is related to blood vessels, and positive and negative values indicate that cell clusters are far away from or close to blood vessels (Galrell, 1996; Pfeiffer et al., 2008).

For the distance between mast cells and the nearest blood vessel, we calculated the cumulative frequency distribution, named [G(d)]. For random points generated by computers, the cumulative frequency distribution was named [G0(d)], and the 95% confidence envelope for [G(d)] − [G0(d)] = 0 was calculated. The hypothesis is no difference between the two functions, G(d) = G0(d), for all distances. If [G(d)] − [G0(d)] is greater than the confidence envelope around [G(d)] − [G0(d)] = 0, mast cells are closer to the vessel than random distribution. If [G(d)] − [G0(d)] is lower than the envelope at approximately 0, mast cells are distributed away from the blood vessel (Guidolin et al., 2017a; Guidolin et al., 2017b).

### Spatial pattern analysis using Spearman’s non-parametric correlation coefficient

Spearman’s non-parametric correlation coefficient (rs) was used to test the distribution pattern of adipocytes and mast cells were in phase or not.

Spearman’s correlation coefficient (rs) = 0 is absence of correlation, > 0 is in-phase, and <0 is out-of-phase. A coefficient 0.7–0.99 is considered strongly correlated, 0.5–0.69 generally correlated, and 0.01–0.49 weakly correlated (Abd Al-Hameeda, 2022). First, the areas where the edge of the fascia was stretched excessively were cut off, then, the whole slide image was divided into multiple square areas of the same size (1,280 μm × 1,280 μm). The number of adipocytes and mast cells was determined in each individual unit. Next, adjacent sample units were added together, which allowed for counts to be obtained for increasing field sizes. Spearman’s coefficients were calculated for different areas of quadrants.

### Multivariable linear regression analysis

Multivariable linear regression models were created by using STATA, and MATLAB was used for ridge regression analysis. Statistical analysis involved using GraphPad Prism 7.0. Data were analyzed by unpaired, two-tailed Student’s *t*-test. *p* < 0.05 was considered statistically significant.

## Results

### Separation of the superficial fascia

**FIGURE 1:**
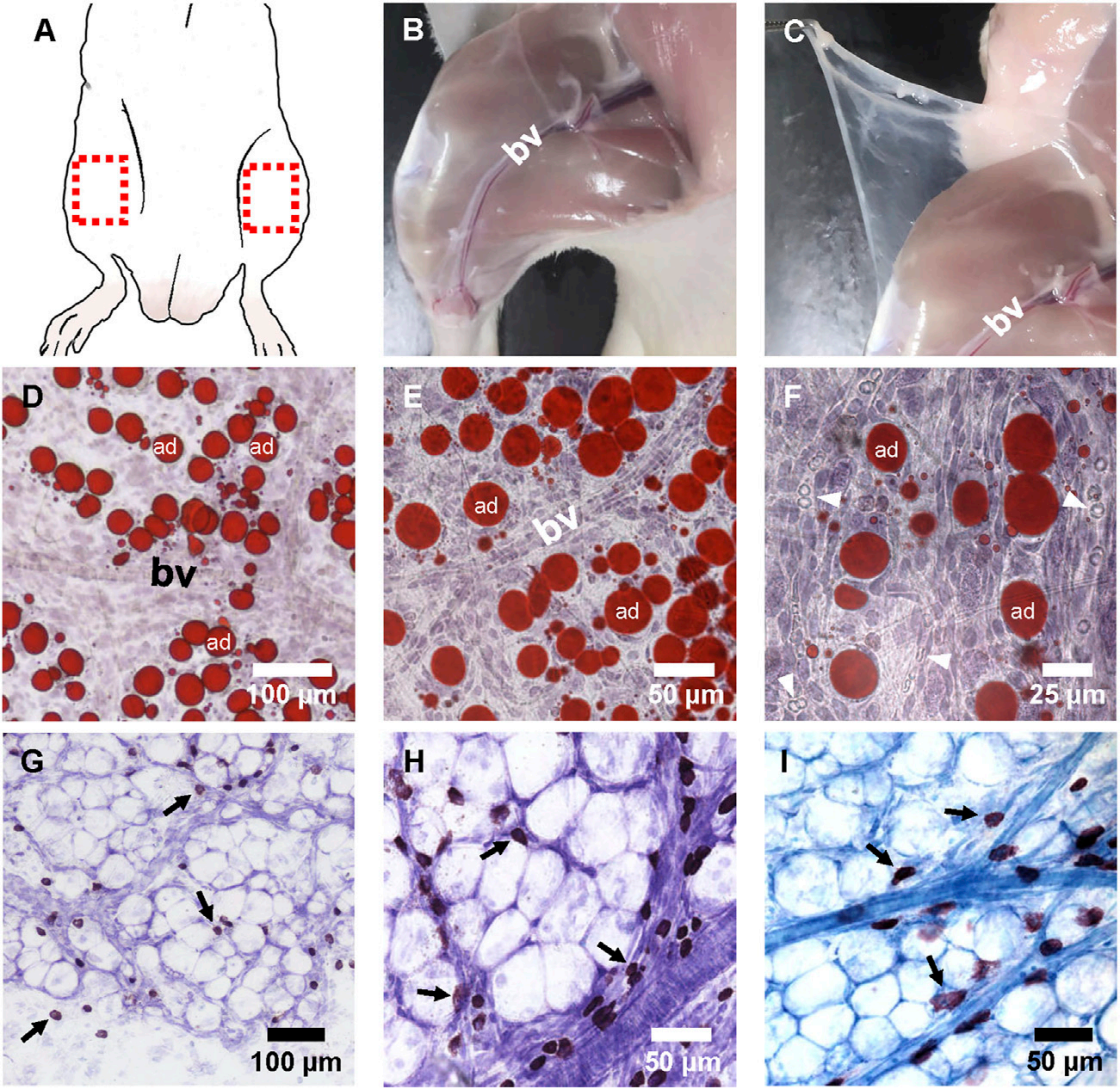
The appearance and histological image of fascia. **(A)** Fascia covered almost all parts of the body. The superficial fascia located above the hindlimb muscles was much easier to separate and was suitable for making whole-mounted fascia patches. The red box shows where the fascia was separated when the patch was made. **(B,C)** Blunt separation and cutting of skin. The superficial fascia appeared to be transparent and covered the skeletal muscles. The blood vessels (bv) of the saphenous artery and vein in the deep fascia of muscles ran under the superficial fascia. **(D–F)** Whole-mounted fascia slides were stained with Oil-red O and hematoxylin at ×100 **(D)**, ×200 **(E)**, or ×400 **(F)** magnification. **(D)** There were lobo-adipocytes (ad) on the superficial fascia, and most of them gathered near the blood vessels. **(E)** Blood vessels ran through the adipose lobules. **(F)** Some red blood cells (arrowheads) with small, round, and biconcave appearance were seen in the vascular lumen. Red blood cells also showed a dumbbell shape in profile. **(G,H)** Whole-mounted fascia slides were stained with thionine at ×200 **(G)** or ×400 **(H)** magnification. Thionine staining of mast cells appeared as a deep brown color. **(G)** Mast cells (arrow) were co-distributed with adipocytes in the superficial fascia. **(H)** Mast cells (arrow) gathered in the cluster around the blood vessels. **(I)** Toluidine blue staining. Mast cells (arrow) were stained as a blue-purple color. Adipocytes and blood vessels could also be distinguished clearly.

The superficial fascia is located between the dermis and hind-limb skeletal muscle groups, and the hind-limb superficial fascia surrounding the lower segment of the saphenous artery and vein was easily separated (Figures 1A–C). After making whole-mounted fascia slides and staining, blood vessels that ran through on the superficial fascia could be observed. These vessels divide the fascial fat into lobules (Figure 1D), and a large number of small blood vessels grew into adipose lobules (Figures 1E,F). Thionine and toluidine blue were used to specifically stain mast cells because mast cells contain a large number of acid-fast granules. With specific staining, abundant mast cells could be seen distributed in the superficial fascia (Figure 1G). Most showed the distribution characteristics of aggregation around blood vessels (Figures 1H,I).

### Distribution of vessels, mast cells and adipocytes in the superficial fascia

**FIGURE 2:**
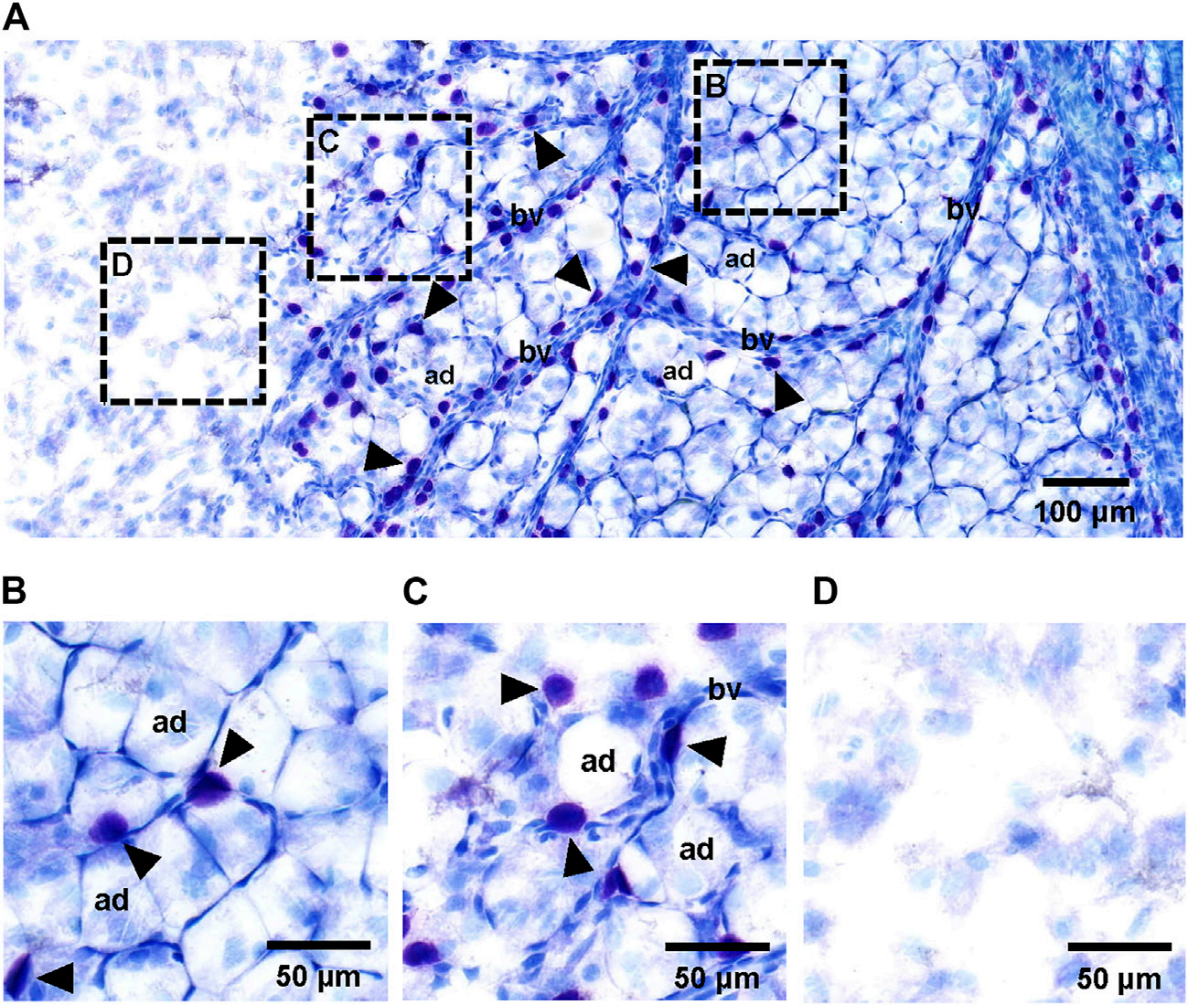
Histology of adipocytes, mast cells and blood vessels in superficial fascia. **(A)** Toluidine blue staining of whole-mounted fascia at ×50 magnification. Small vessels (bv) ran through the superficial fascia. Primitive adipose lobule area filled with large number of adipocytes (ad) was distributed around blood vessels. Adipocytes were always accompanied by a number of mast cells (arrowheads) in the superficial fascia. **(B–D)** Toluidine blue staining of whole-mounted fascia at ×100 magnification. Histological whole slide image of fascia could be divided into three areas: primitive adipose lobule area **(B)**, scattered adipocyte area **(C)** and non-fat blank area **(D)**. The primitive adipose lobule area was filled with a large number of adipocytes and some mast cells. The scattered adipocyte area containing a number of sporadic adipocytes. In this area, the distribution of cells was sparse, with more space for mast cells; therefore, the number of mast cells may be slightly more than that in primitive adipose lobule area. In the non-fat blank area without blood vessels and mast cells, there were no adipocytes.

The superficial fascia, which was separated from the hindlimbs of 4-week-old rats, was stained with toluidine blue. Toluidine blue staining is commonly used to identify mast cells, and the stained mast cells had a blue–purple appearance (Figure 2A). The distribution of adipose lobules along blood vessels was clearly observed, whereas many blue–purple cells accumulated around the vessels (Figures 2B,C). In the absence of blood vessels, there were very few mast cells or adipocytes (Figure 2D).

**FIGURE 3:**
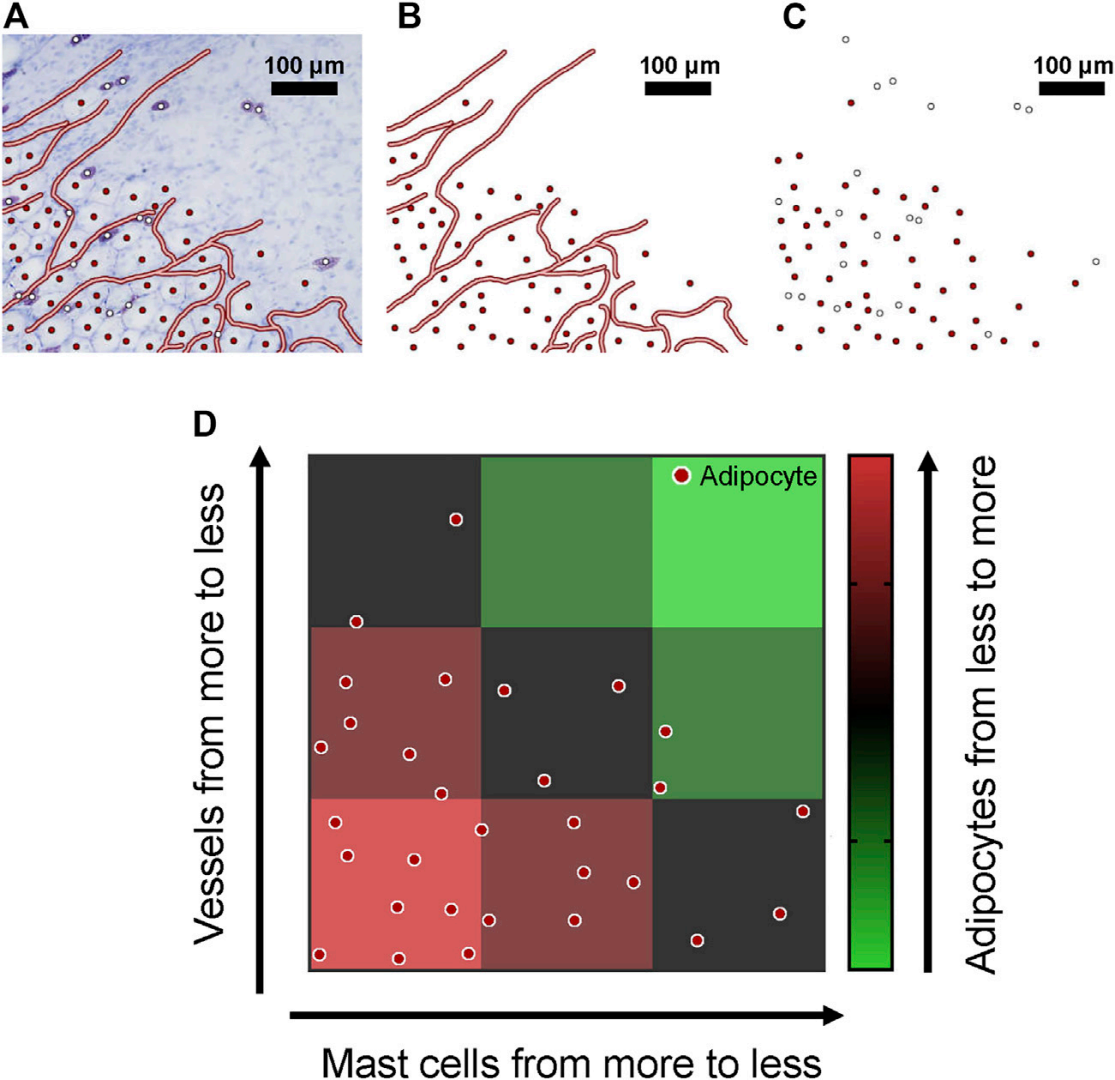
Blood vessels, mast cells and adipocytes were concentrated in the superficial fascia. **(A)** Toluidine blue staining of whole-mounted fascia from 4-week-old rats; blood vessels, adipocytes and mast cells are marked. **(B)** Only blood vessels and adipocytes are shown; adipocytes were always distributed near blood vessels. **(C)** When only mast cells and adipocytes are shown, mast cells and adipocytes were co-distributed in the superficial fascia. **(D)** The spatial distribution of blood vessels, mast cells and adipocytes is shown by a schematic diagram. With the decrease in number of mast cells and vascular density, the number of adipocytes decreased significantly.

Mast cells and adipocytes co-distributed in the superficial fascia, always localized along with blood vessels in the superficial fascia. Scattered adipocytes grew into adipose lobules along blood vessels. At the edge of the adipose lobules, growth trends could still be seen. The vascular skeleton, adipocytes and mast cells were marked with lines and dots in images of the edge of adipose lobules (Figure 3A). The red dots indicate adipocytes and white dots mast cells. Then, blood vessels with adipocytes and mast cells with adipocytes are shown separately (Figures 3B,C). Most obviously, adipocyte density was affected by blood vessels and mast cells. With an increase in mast cells and vascular density, the number of adipocytes also increased (Figure 3D). This observation suggests that mast cells, adipocytes and blood vessels are closely related. So next, we analyzed the relation between the three structures by quantitative calculation.

The nearest distance between cells and blood vessels could be measured in the tissue photos containing mast cells and blood vessels, which revealed the distribution information about the mast cells and blood vessels. Then the effects of the number of mast cells and distance from blood vessels on the distribution of adipocytes were analyzed quantitatively by spatial pattern analysis. Multivariable linear regression analysis was used to exclude the influence of the distribution relation between mast cells and blood vessels on the analysis of adipocyte distribution.

### The disordered distribution of the vascular network

**FIGURE 4:**
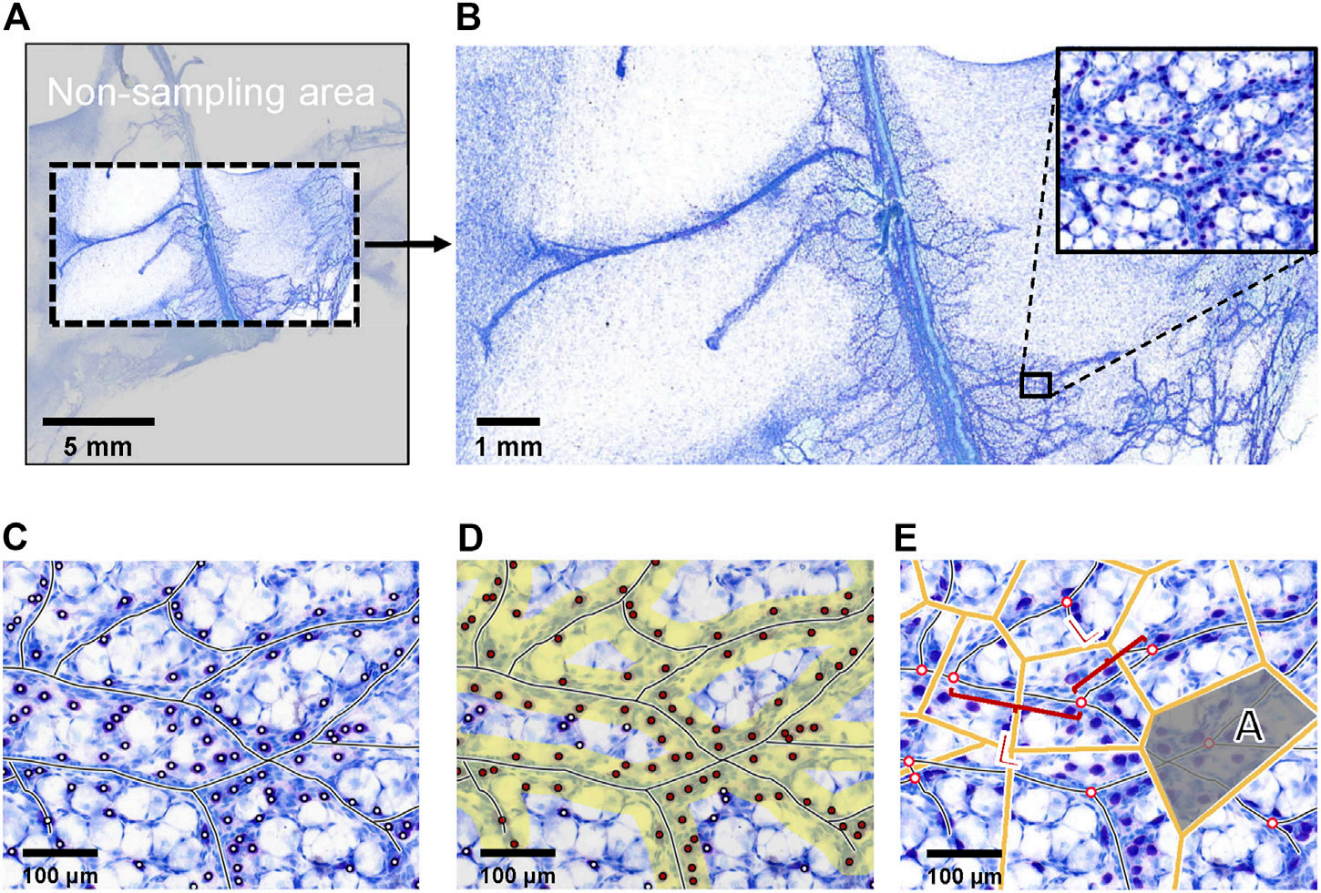
Example of picture processing steps. **(A)** The picture of Toluidine blue staining of whole-mounted fascia from 4-week-old rats was the object of program analysis. Because fascia is a tissue with high ductility, the edge of the superficial fascia was easy to pull, so that the edge of the patch was not analyzed. **(B)** Because Toluidine blue stain has low color contrast for mast cells, adipocytes and blood vessels, it was not suitable to input programs directly. We needed to pre-label cells and blood vessels. **(C)** The blood vessels and mast cells were marked on the panoramic image. **(D)** The pictures of blood vessels and cells were input into the program. The yellow area represents the area within the defined distance from the vessel. Red points indicate the cells that were within a specified distance. The program automatically counted the number of red dots in the yellow area to obtain the number of cells within a certain distance of the vessel. **(E)** Branching points marked by the program, with its binary skeleton. The length of the skeleton segments between two branching points was L_*j*_. The corresponding Voronoi diagram of branching points generated by the program. The size of each Voronoi cell was A_*i*_; subscript *i* indicates the *i*th Voronoi cells.

Two types of order were considered: positional order and topological order. After excluding the overstretched portion of the fascia patch (Figures 4A,B), by using image processing software, the vascular skeleton was marked on the image of superficial fascia (Figures 4C,D), and the length between the skeleton branches was measured as L_*j*_. Then, a Voronoi diagram was generated, and the area of each region named A_*i*_ was measured (Figure 4E). The subscript indicates the *i*th Voronoi cells. The positional order was associated with the arrangement of the vascular network and was quantified as the parameter SD. In superficial fascia separated from the hind limbs of 4-week-old rats (*n* = 3), the SD was 0.7732. The topological order was formulated as TD to characterize the homogeneity of branching occurrence along the structure. In our study, TD was 0.5600. Because both parameters were >0, as mentioned above, the vascular network was determined as a disordered distribution in the superficial fascia.

### Mast cells are distributed along the blood vessels in superficial fascia

**FIGURE 5:**
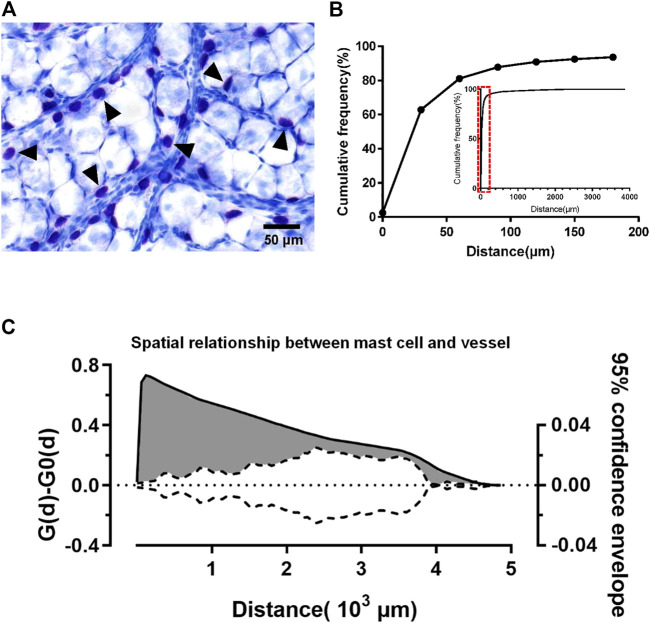
Mast cells were distributed along the blood vessels. The spatial pattern of adipocytes and mast cells in superficial fascia (three rats at 4 weeks old) was analyzed using the method described by Guidolin et al. The distance between the mast cell to the nearest blood vessel was measured by the program. **(A)** Toluidine blue staining of whole-mounted fascia; mast cells (arrowheads) stained purple can be seen near the blood vessels. **(B)** Cumulative adipocyte frequency of distance from mast cells to the nearest blood vessel from rats (*n* = 3) at age 4 weeks 62.89% mast cells were within 30 μm of the vessel. More than 90% of the mast cells were less than 150 μm away from the vessel. According to the small graph of relative frequency, mast cells distributed most at 30 μm. **(C)** Analysis of the spatial relationship between mast cells and blood vessel. The solid line corresponds to the left coordinate axis, indicating the difference between the observed distribution of mast cell-to-vessel distance [G(d)] in the superficial fascia and the estimated distribution [G0(d)] generated by the program. The dotted line corresponds to the coordinate axis on the right, indicating the 95% confidence envelope for [G(d)] − [G0(d)] = 0. At different distances, the values of solid lines are larger than dashed lines (gray area), which suggests that mast cells were closer to the vessels than random distribution points. The distribution of mast cells along blood vessels can be shown.

The superficial fascia separated from the hind limbs of 4-week-old rats was made into whole-mount slides. In the slides of superficial fascia stained with toluidine blue, mast cells were mostly distributed around blood vessels (Figure 5A). To verify this distribution pattern of mast cells, we scanned slides to produce pictures and the program was used to measure the distance from mast cells to the nearest blood vessel. Approximately 62.89% of mast cells was <30 μm away from vessels (Figure 5B). As compared with the random point patterns, this distance was much shorter, and the difference was statistically significant. In the confidence interval curve, the area beyond the curve indicates that the spatial distribution of mast cells was much closer to the vessel than a random distribution (Figure 5C). This evidence suggests that mast cells are clustered along blood vessels.

### Adipocytes are also distributed along blood vessels in superficial fascia

**FIGURE 6:**
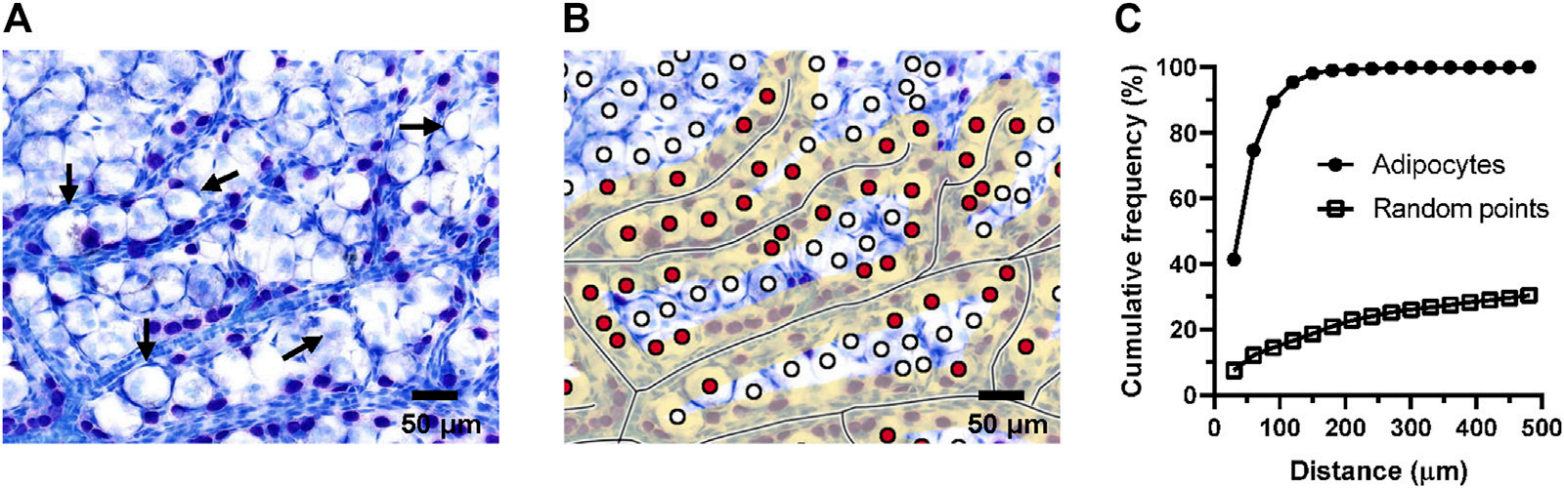
Adipocytes were distributed around the vessels. For the superficial fascia of 4-week-old rats, the number of adipocytes within a certain distance from the blood vessel were counted by the program. The fascia was sampled from three rats. **(A)** Toluidine blue staining of whole-mounted fascia at ×50 magnification. Adipocytes (Long arrow) were always distributed near the blood vessels. **(B)** Toluidine blue staining of whole-mounted fascia marked with blood vessels and adipocytes. The yellow area represents the area within the defined distance from the vessel. Red points were the adipocytes that were within the given distance. **(C)** The cumulative frequency curve of adipocytes was higher than the random point, which indicated that the number of fascial adipocytes appeared greater than random points at the same distance. Adipocytes were closer to blood vessels, which suggests that adipocytes were distributed around the vessels. About 74.74% adipocytes were close to blood vessels by 60 μm, equivalent to the diameter of two adipocytes. 89.44% of the adipocytes are less than 90 μm away from the blood vessels.

Because adipocytes have a larger size, the above method may not be adequate for exploring the relation between adipocytes and blood vessels, so we adjusted the criteria for screening samples by statistical methods to adapt to the larger diameter of adipocytes. The diameter of adipocytes in superficial fascia from 4-week-old rats was approximately 32 μm. Thus, we took the sum of the diameters of two or three adipocytes as the criterion, approximately 60 and 90 μm. We counted the number of cells within a determined distance by using the computer program (Figures 6A,B). After analyzing the pictures of the slides of superficial fascia, 74.74% of adipocytes were close to blood vessels at 60 μm and approximately 89.44% of adipocytes were at 90 μm. Hence, adipocytes were close to vessels. The cumulative frequency curve of adipocytes was higher than the random point generated by computers. The number of adipocytes was more than that for random distribution around the blood vessels, which suggests that adipocytes were also distributed along the vessels (Figure 6C).

### Adipocytes and mast cells are co-distributed in superficial fascia

**FIGURE 7:**
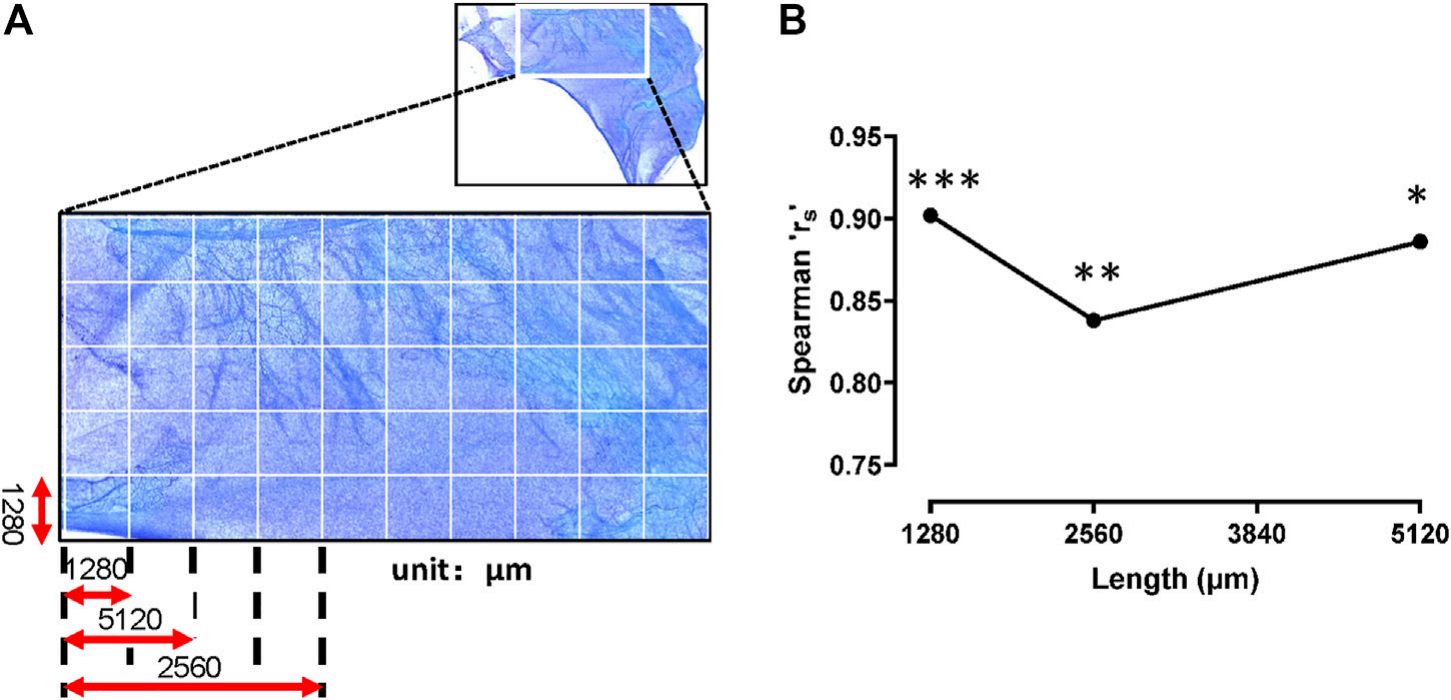
Mast cells and adipocytes were distributed in phase. **(A)** The schematic diagram. The whole slide image of fascia (upper panel) was segmented into a series of adjacent sample areas with gradually increasing field sizes. The initial field size of the smallest unit was set up with the width and length of 1,280 μm × 1,280 μm. Mast cells and adipocytes in the sample areas were counted. **(B)** Spearman correlation coefficient (rs) was used to analyze the spatial correlations between adipocytes and mast cells in the increasing sample areas. The rs values were all >0.8 at field sizes between 1 μm × 1,280 μm × 1,280 μm and 3 μm × 1,280 μm × 1,280 μm, indicating that the spatial distribution of the two cell types were in phase. ***, *p* < 0.001, **, *p* < 0.01, *, *p* < 0.05.

Because adipocytes and mast cells were both distributed along the blood vessels, correlation analysis was needed to determine whether these two cell types were co-distributed. Spearman’s correlation coefficient (rs) was used to analyze the degree of spatial association between the two cell types. This coefficient shows the strength and direction of the relation between two objects. Its value is between −1 (negative relation) and 1 (positive relation); 0 indicates no correlation between the two. A coefficient of 0.7–0.99 is generally considered to indicate a strong correlation. In this study, the Spearman’s correlation coefficient (rs) was >0.7 under different areas. We found significant positive correlations between adipocytes and mast cells from 1 μm × 1,280 μm × 1,280 μm to 3 μm × 1,280 μm × 1,280 μm (Figure 7). Therefore, the distributions of two cell types were positively correlated and were spatially in-phase in the superficial fascia.

### Multivariable linear regression analysis for identifying the effect of mast cells and blood vessels on adipose differentiation

As mentioned earlier, we showed that both mast cells and adipocytes were distributed along the blood vessels in the superficial fascia. In addition, mast cells and adipocytes were distributed in-phase. Therefore, we believed that blood vessels and mast cells have certain effects on adipocyte differentiation, which we hoped to verify by mathematical analysis.

**FIGURE 8:**
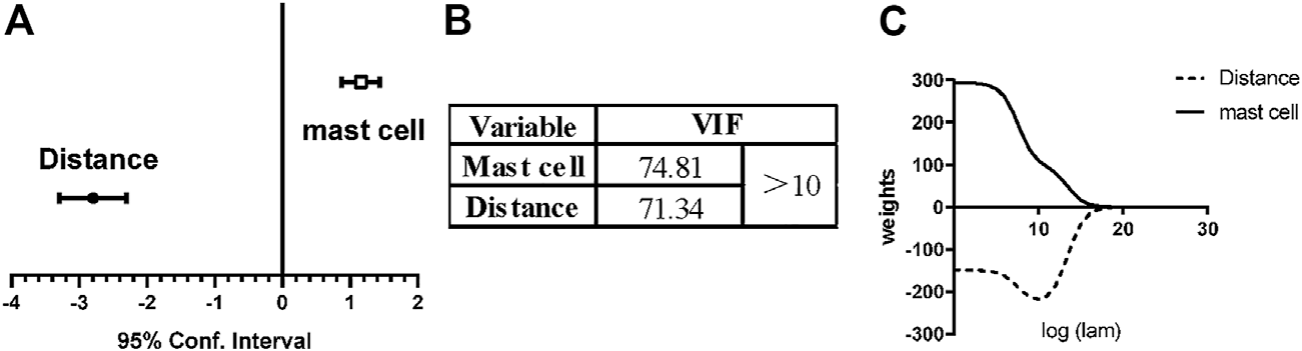
Linear multi-regression analysis and ridge regression analysis for vessels, mast cells and adipocytes. To explore the effect of blood vessels and mast cells on adipose differentiation, we used linear multi-regression analysis and ridge regression analysis. **(A)** For linear multi-regression analysis, the confidence interval of the distance from vessels was [−3.29, −2.30], <0, which means that the closer to the vessel, the more adipocytes. The confidence interval of the number of mast cells was [0.88, 1.43], >0, which means that the adipocyte number increased with the existence of mast cells. **(B)** The variance inflation factor (VIF) of the distance from vessels and the number of mast cells, both >10. This model exhibits multicollinearity. **(C)** Ridge regression analysis was used to eliminate multicollinearity. The coefficient of distance from blood vessel was always <0 and coefficient of the number of mast cells was always >0. Therefore, the above conclusion is still valid, and the effect of mast cells promoting adipose differentiation was independent of blood vessels.

First, we used multivariable linear regression analysis to derive the relations among mast cells, blood vessels and adipocytes. The confidence interval of the distance from vessels was [−3.29, −2.30], whereas the confidence interval of the number of mast cells was [0.88, 1.43] (Figure 8A). In other words, the closer to blood vessels and the more mast cells are present, the more adipocytes are present.

However, the variance inflation factor (VIF) of the distance from vessels and the number of mast cells was 71.34 and 74.81, respectively, and both values were >10 (Figure 8B), so multicollinearity occurred for these two independent variables, which harmed the results and the mathematical model. As predictor variables, the distribution of mast cells and adipocytes are both highly correlated with the distribution of vessels, which is why multicollinearity occurs in regression analysis. Therefore, we next used ridge regression to eliminate multicollinearity. After ridge regression analysis, the coefficient of mast cell number was always >0, and the coefficient of distance from vessels was always <0 (Figure 8C). The relations among mast cells, blood vessels and adipocytes still held. With mathematical methods, we again concluded that the spatial distribution of mast cells was related to adipocytes. With increasing number of mast cells, the number of adipocytes will increase, and these adipocytes can even grow into primitive adipose lobules.

### Analysis of the relation between mast cells, adipocytes and blood vessels

To quantitatively show the relations among mast cells, adipocytes and blood vessels, we first simplified the structure of cells and blood vessels into points and lines and analyzed the spatial association of different units from the aspects of lines and lines, points and points, points and lines etc. The Guidolin et al. statistical analysis methods from epidemiology and ecology were adopted to quantify the distribution pattern and correlations among blood vessels, adipocytes, and mast cells. Image processing software and self-developed computer programs were used to analyze the images of whole-mounted fascia, and the relevant data were measured automatically. The analysis based on the Voronoi model indicated that the vascular network was non-uniformly distributed. Overall, 81.16% of mast cells and 74.74% of adipocytes were distributed within 60 μm of blood vessels on the fascia with an average area of about 3.75 cm^2^. The Spearman correlation coefficient (rs) of >0.7 showed the co-distribution of the two types of cells under different fascia areas. Moreover, the ridge regression analysis further revealed the spatial correlation among blood vessels, adipocytes and mast cells.

## Discussion

The morphological distribution of mast cells and adipocytes showed an interaction between the cells in a physiological state. However, adipose tissue sections used in conventional morphological studies can only show the distribution pattern of cells in one section. The vascular network always runs through various tissues, so a single section is not sufficient to show its structural distribution. As we have shown before, in this study, both mast cells and adipocytes were distributed around blood vessels, so blood vessels are an important interference factor in analyzing the relation between mast cells and adipocytes. However, the traditional method of histological sectioning has difficulty showing the distribution of vascular and mast cells at the same time. Tissue slices can only show the relation between the vasculature and cells on one flat surface; more complete information is needed.

In the present study, we selected superficial fascia separated from the hind limbs of rats for analysis. The superficial fascia is suitable for studying blood vessels and adipocytes. According to the structural characteristics of its flaky film, the distribution characteristics of vessels and cells in the fascia *in vivo* could be retained to the greatest extent through patches in this study. With panoramic scanning, we could acquire a complete image of the fascia slide with a single-cell distribution. The vascular network and cells are distributed on the fascia sheet, which allows for measuring the shortest distance between the three components and analyzing their relation. In our previous study, we showed that mast cells and adipocytes in superficial fascia occur in clusters and their distribution was positively correlated with each other. More specifically, the variance-mean ratio and Morisita index indicated that mast cells and adipocytes were distributed in clusters, and Spearman’s correlation coefficient revealed the correlation between their distributions (Zhang et al., 2019). Considering the influence of blood vessels on the distribution of the two types of cells, blood vessels must be added in the analysis as a possible factor leading to the aggregation of mast cells and adipocytes.

The aim of this study was to explain the relation between blood vessels, mast cells and adipocytes by using quantitative analysis. We also aimed to build a credible quantitative analysis model by adopting the Guidolin et al. statistical analysis method from epidemiology and ecology. The simplest and most intuitive way is to build the linear regression equation. We calculated the VIF, a parameter that can reveal the collinearity of independent variables and revealed multicollinearity in the distribution of the two cell types. Because of multiple collinearity problems among mast cells and adipocytes, it was not reasonable to build linear equations directly. Therefore, we used ridge regression analysis of the data. The effect of mast cells on the distribution of adipocytes could be judged according to positive and negative correlation coefficients. By mathematical treatment, we have shown that the distribution of mast cells and adipocytes is affected by blood vessels, but there was a certain correlation between them, so mast cells and adipocytes were co-distributed. Our laboratory previously showed that the superficial fascia is the origin of adipocytes (Su et al., 2016). The correlation coefficient between the spatial distribution of mast cells and adipocytes increased significantly during the periods of rat growth, which was consistent with the increasing number of adipocytes trend in fascia (Zhang et al., 2019). Therefore, the co-distribution of mast cells and adipocytes may be not only a spatial coincidence but mast cells may also participate in the growth of fat, so that the more mast cells distributed, the more adipocytes are increased in number. Primary preadipocytes from fascia cultured with supernatants of mast cells stimulated adipogenic differentiation (Chen et al., 2021). From the above histology and cytology results, we speculate that mast cells can promote adipogenesis.

**FIGURE 9:**
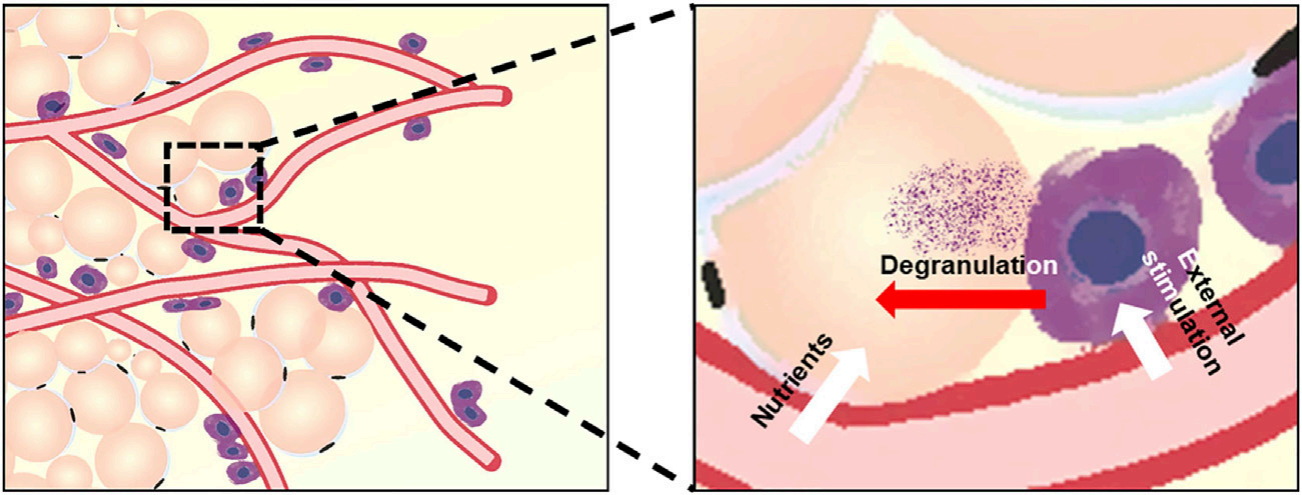
Model for the relation of vascular vessels, mast cells, and adipocytes in superficial fascia. Blood vessels run through the superficial fascia and show disordered distribution, whereas adipocytes and mast cells show obvious distribution patterns around blood vessels. The distribution pattern of cells is related to their physiological function, and we have shown this by quantitative analysis.

As shown in Figure 9, blood vessels are interspersed in the superficial fascia, and mast cells are distributed next to the blood vessels. The unique distribution pattern of mast cells may be related to its physiological function. Mast cell precursor cells circulate in the blood and migrate from blood vessels. Mast cells can discern a variety of infectious agents near the blood vessels, which may help mast cells contact the external substances and function (Yong, 1997). Vessels and mast cells together constitute a microenvironment suitable for directional differentiation of precursor cells into adipocytes. Blood vessels are necessary structures for tissue to exchange nutrients and waste between the blood and cells, which may be why adipocytes grow close to blood vessels, especially when the main function of adipose tissue is to store energy. The relation between mast cells and adipocytes may be that some substances involved in the degranulation of mast cells promote the proliferation and differentiation of adipocytes. In recent studies, our laboratory found that heparin in mast cell degranulation products can significantly promote fat differentiation and that mast cells are indeed related to adipogenesis (Chen et al., 2021).

By extending the Guidolin et al. statistical analytical method, we developed a powerful tool to analyze the spatial relation between different kinds of cells and vessels, even quite different cell sizes. The analysis procedure may be used to simplify the structure of cells and blood vessels into points and lines for analyzing the spatial relation of different units from the aspects of lines and lines, points and points, points and lines etc. With the program written in MATLAB, the required data can be extracted directly from the input image. Then with the preset formula calculation, the spatial distribution mode of each component can be analyzed.

In summary, this study quantitatively showed the relations among mast cells, adipocytes and blood vessels. We found a positive correlation between the distribution of blood vessels and the two cell types and between that of the two cell types themselves, so the three tend to co-distribute. This phenomenon was also consistent with the respective physiological functions of the structures. Therefore, we successfully quantified the spatial distribution of the three structures and also constructed a complete and effective analysis method. This method includes the analysis of linear structure distribution, point structure distribution, and the relation between point and line distribution and is suitable for histological and morphological studies of fascia and related soft connective tissues.

## Data Availability

The original contributions presented in the study are included in the article/supplementary material, further inquiries can be directed to the corresponding author.
